# Correlation Between Nasal Anatomical Variants and SNOT-22 in Patients Affected by Odontogenic Sinusitis: A Retrospective Study

**DOI:** 10.3390/jcm14072337

**Published:** 2025-03-28

**Authors:** Federico Sireci, Filippo Cascio, Francesco Lorusso, Francesco Gazia, Angelo Immordino, Salvatore Gallina, Valerio Campofiorito, Andrea Comparetto, Ignazio Gerardi, Francesco Dispenza

**Affiliations:** 1 Otorhinolaryngology Section, Department of Precision Medicine in Medical, Surgical and Critical Care (Me.Pre.C.C), University of Palermo, Via del Vespro, 133, 90127 Palermo, Italy; federicosireci@hotmail.it; 2 Department of Otorhinolaryngology, Papardo Hospital, Contrada Papardo, 98158 Messina, Italy; filippocascio85@gmail.com; 3 Otorhinolaringology Section, Department of Biomedicine, Neuroscience and Advanced Diagnostics, AOUP Paolo Giaccone, University of Palermo, Via del Vespro, 133, 90127 Palermo, Italy; dott.francescolorusso@gmail.com (F.L.); angelo.immordino182@gmail.com (A.I.); salvatore.gallina@unipa.it (S.G.); valeriocampofiorito@gmail.com (V.C.); andrea.comparetto@libero.it (A.C.); ignazio.gerardi@gmail.com (I.G.); francesco.dispenza@gmail.com (F.D.)

**Keywords:** SNOT-22, odontogenic sinusitis, anatomical variants, endoscopic sinus surgery

## Abstract

**Objectives**: The aim of this study is to analyze the correlation between the nasal anatomical variants and the clinical and radiological features of odontogenic sinusitis to demonstrate their possible involvement in the genesis of the disease. **Methods**: This is a retrospective multicentric study of 70 patients with odontogenic sinusitis (OS). Before surgery, all patients performed the classic and mini 22-item Sino-Nasal Outcome Test (SNOT-22 and SNOT-8) and Lund–Mackay Score (LMS). Each nasal anatomical anomaly was collected, including paradoxical middle turbinate (PMT), chonca bullosa (CB), and nasal septal deviation (NSD) on the same side as the sinusitis. **Results**: The patients presented a significantly higher SNOT-22 and SNOT-8 score only in case of association with NSD and PMT/CB. Logistic regression showed that the variables were significantly associated (*p* = 0.02 and 0.04). No significant correlation was found between LMS and anatomical variations. **Conclusions**: This study showed that nasal anatomical variables are correlated with SNOT-22 and SNOT-8. Having a combination of NSD and CB or PMT, on the same side as an OS, is related to a worse quality of life in patients affected by this disease. Regarding the radiological features of OS, no significant correlation was found between LMS and anatomical variations.

## 1. Introduction

Odontogenic sinusitis (OS) is an inflammation of the maxillary sinus with or without involvement of other sinuses, which recognizes as primum movens a variety of dental pathologies such as the following: endodontitis, periodontitis, and oroantral fistula [1]. OS occurs as a result of Schneiderian membrane perforation through dentoalveolar pathology. The most common cause of OS is iatrogenic (55.97%), including incorrectly performed sinus lift procedures, dental implants with dimensions and insertion axes not adapted to the individual clinical features, foreign bodies (perforations during endodontic treatments, overfilling of root canals beyond the apex with filling materials such as zinc oxide eugenol or gutta percha), and dental extractions with or without the displacement of a root fragment into the sinus cavity [2].

Some studies have shown that OS represents about 25–40% of the cases of rhinosinusitis, especially unilateral [3].

It often presents non-specific symptoms characterized by nasal obstruction, anterior or posterior rhinorrhea, facial pain or pressure, and hyposmia or anosmia [4], most often unilateral. If not addressed correctly, this condition can lead to serious complications, such as the extension of the disease to the structures of the skull or of the orbital cavity [5].

In cases of OS, properly addressing the underlying dental infection and associated sinusitis results in resolution in over 90% of patients. Notably, the management of OS differs significantly from that of non-odontogenic rhinosinusitis. Key considerations in OS management include the presence of complicated OS (such as extrasinus infectious spread), the identification of treatable dental pathology, and the relative symptom burden of dental issues versus sinusitis [4].

OS should be incorporated into the broader context of sinonasal diseases, emphasizing the need for multidisciplinary collaboration between otolaryngologists and dental specialists to enhance clinical outcomes, advance research, and improve education. Key challenges include deepening the understanding of dental and sinus pathophysiology, establishing universally accepted diagnostic criteria, and optimizing integrated treatment pathways [5].

Not all patients with dental diseases develop sinusitis symptoms, and the nasal predisposing factors, especially the nasal anatomical variants, have not been studied extensively. Globally, there is a high variation in the reported prevalence rates of nasal septal deviation (NSD) ranging from 26% to 97%, which can be explained by the extent of deviation considered in the reported studies [6]. In the largest case series in the literature, middle concha bullosa (CB) was detected in 1402 (44.74%) of cases [7]. Paradoxical middle turbinate (PMT) is commonly encountered. Prevalence ranges from 9 to 34% [3,6,7]. When present it is bilateral in 40–80% of cases [8,9].

NSD and nasal spurs can lead to lateral narrowing of the middle turbinate and compression of the middle meatus, resulting in impaired ventilation and facial pain. Additionally, a septal spur may compress the inferior turbinate, which is innervated by branches of the maxillary nerve, causing intermittent pain that varies with the degree of turbinate hypertrophy.
CB is defined as the pneumatization of the middle turbinate. It can obstruct the air passage by blocking the middle nasal meatus or cause mucosal edema, inflammation, drying, and headaches by impeding the ethmoid infundibulum.
PMT refers to an abnormal curvature of the middle turbinate, in which the convex surface faces laterally instead of medially. This abnormality can obstruct the drainage pathway of the middle meatus.

The aim of this study is to study the correlation between the nasal anatomical variants and the clinical and radiological features of OS to demonstrate their possible involvement in the genesis of the disease.

## 2. Materials and Methods

### 2.1. Study Design and Data Collection

This is a retrospective multicenter study including 70 patients with OS, treated in the “Paolo Giaccone” University Hospital in Palermo and the “Papardo” Hospital in Messina, between 2016 and 2022 (Table 1). The mean age is 48.15 ± 12.84, with 45 males and 25 females. The study was conducted in accordance with the Declaration of Helsinki and approved by the Ethics Committee of Palermo 1 (protocol n° 11 of 22 April 2024).

Regarding the study, the inclusion criteria are as follows:

- Adults over 18 years of age;
- Diagnosis of OS with CT scan or nasal endoscopy and clinical history (OS appears due to inflammation of the mucosa of the maxillary sinus characterized by two or more symptoms, one of which must be nasal obstruction or nasal discharge associated with pain or facial pressure and/or reduction or loss of smell for at least 12 weeks as a result of Schneiderian membrane perforation through dentoalveolar pathology [2]);
- Patients who performed ESS. Patients who are candidates for surgery are those who have not responded to conservative medical therapy or odontogenic treatments and have suffered a relapse of disease.

Patients who had already undergone nasal surgery and those affected by Churg–Strauss syndrome, Kartagener syndrome, and cystic fibrosis were excluded from the study.

**Figure 1:**
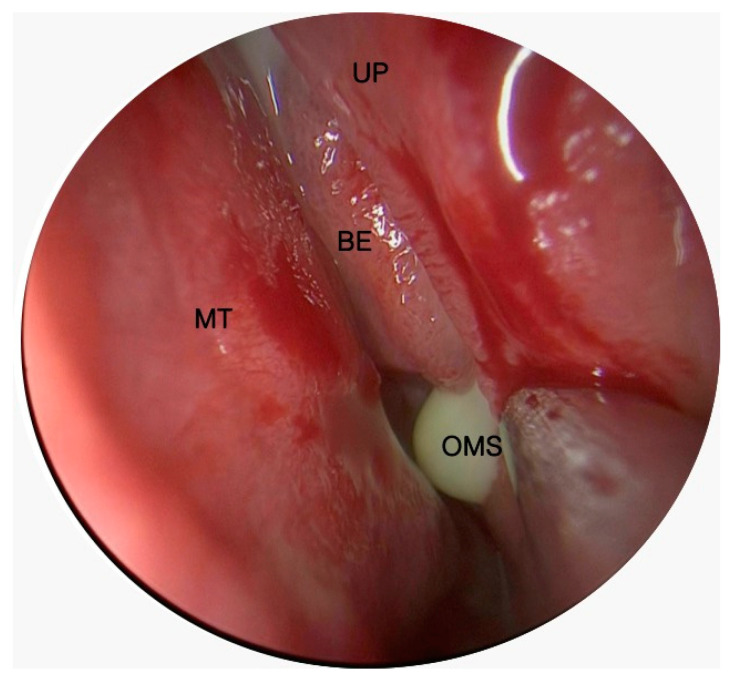
Intraoperative photo of odontogenic left maxillary sinusitis. MT: middle turbinate; BE: bulla ethmoidalis; UP: uncinate process; OMS: ostium of the maxillary sinus.

We included all patients undergoing endonasal endoscopy to evaluate the presence of purulent secretion in the osteomeatal complex or edema of the uncinate process (Figure 1).

**Figure 2:**
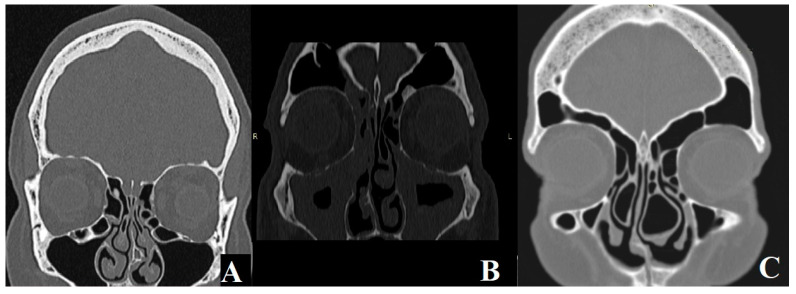
(**A**) Nasal septal deviation on the left side. (**B**) Paradoxical Middle Turbinate on the left side. (**C**) Bilateral Chonca Bullosa.

Information on any anatomical abnormalities affecting the nasal structure was also collected, including paradoxical middle turbinate (PMT), chonca bullosa (CB), and nasal septal deviation (NSD) on the same side as the sinusitis. An example of nasal anatomical variants analyzed by us is presented in Figure 2.

**Figure 3:**
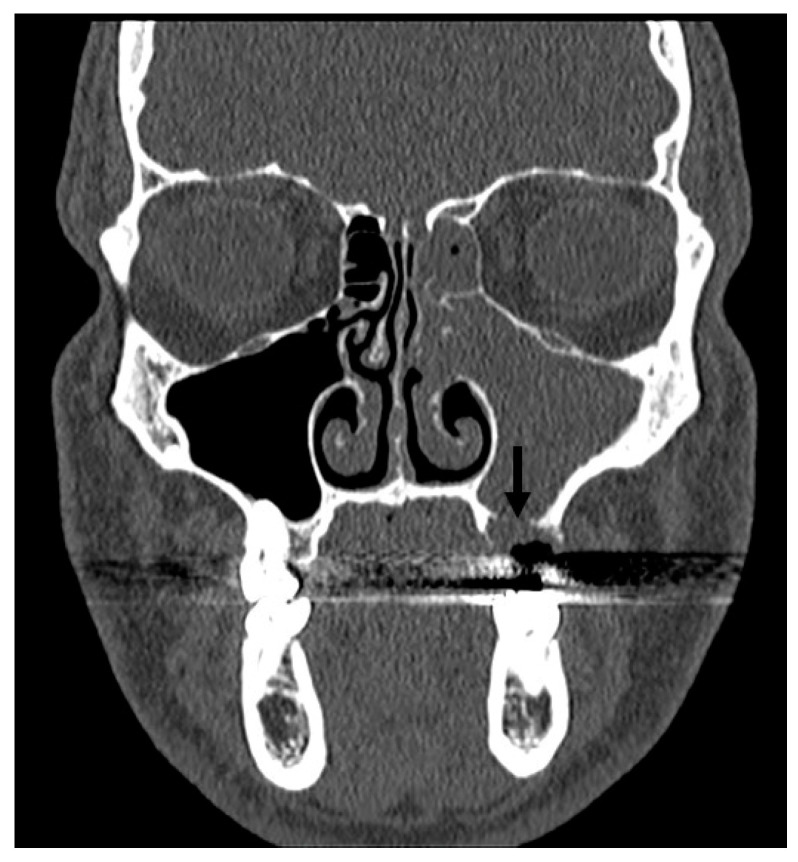
CT scan of odontogenic left maxillary sinusitis. OAF: oroantral fistula (arrow).

After a preoperative CT scan (Figure 3), all the selected patients underwent endoscopic sinus surgery (ESS) [10].

Before surgery, all enrolled patients performed the 22- and 8-item Sino Nasal Outcome Test (SNOT-22 and SNOT-8 questionnaire) and Lund–Mackay Score (LMS) test to analyze which paranasal sinuses were involved. The type of surgery performed depended on the extent of the pathology, and the type of anatomical variant found. Correction of NSD, PMT, or CB was performed if it altered the ventilation of the affected maxillary sinus.

The nasal pack was used only in cases of septoplasty and, in such cases, removed 2 days after surgery. A therapy was recommended, which included an amoxicillin/clavulanate combination or ceftriaxone, saline nasal irrigation, and the application of gomenolato oil spray for seven days.

### 2.2. -22 and -8 Items Sino-Nasal Outcome Test (SNOT-22 and SNOT-8)

SNOT-22 is a 22-item questionnaire used to measure the outcomes of sinonasal conditions and surgical treatments (score range, 0–110) [11,12,13]. It was derived from the SNOT-20, with two additional questions that aim at measuring nasal blockage and sense of taste/smell. A higher total score on the SNOT-22 indicates a greater impact of CRS on health-related quality of life (HRQoL). SNOT-8 is a questionnaire that includes only the following rhinological items of the classic SNOT-22: need to blow nose, sneezing nasal secretions, nasal obstructions, smell and taste, cough, retronasal drip, thick nasal secretion, and facial pressure pain [14]. SNOT-22 is a questionnaire with international validity, used as an indicator of QoL in patients who must start biological therapy (dupilumab, omalizumab, mepolizumab, etc.). For this reason, the questionnaire is considered highly valid in the literature, and we used it in this study.

### 2.3. Lund-Mackay Score (LMS)

The Lund–Mackay staging system scores each sinus (anterior ethmoid, posterior ethmoid, maxillary, frontal, and sphenoid sinuses) according to the following scale: 0 (no opacification), 1 (partial opacification), or 2 (complete opacification). The osteomeatal complex is scored as 0 (not occluded) or 2 (occluded) [15].

### 2.4. Statistical Analysis

Statistical analysis was performed using SPSS 25.0 (IBM SPSS Statistics, New York, NY, USA). Data are presented as means with standard deviations or numbers and percentages. Data normality was assessed using the Kolmogorov–Smirnov test of normality. Logistical regression was used to evaluate the correlation between the anatomical anomaly and SNOT-22, SNOT-8, and Lund-Mackay. Statistical significance was attributed to *p*-values ≤ 0.05.

## 3. Results

Seventy patients were included, with a mean age of 48.15 (± 12.84) years. All patients underwent ESS and, in particular, all of them underwent maxillary senotomy, 25 frontal senotomy, 53 plastic surgery of the middle turbinate, 32 anterior ethmoidectomy, and 32 septoplasties. Forty-two of them required tooth extraction and oroantral fistula closure. During the operation, a microbiological sample was collected in 12 patients and was found to be positive for E.Coli, S.Aureus, and coagulase-negative Staphylococcus; in the remaining cases, the microbiological examination was negative.

The patients presented a significantly higher SNOT-22 and SNOT-8 score only if the OS was associated with CB or PMT and NSD. Logistical regression showed that the variables were significantly associated (*p* = 0.02 and 0.04). The isolated presence of a NSD, CB, or MTP was not correlated with an increase in the SNOT-22 or the SNOT-8 scores (*p* > 0.05) (Table 2 and Table 3).

Furthermore, we analyzed the correlation between the LMS and anatomical variation. No significant correlation was found (Table 4). Six months after surgery, patients performed SNOT-22 and SNOT-8; the results indicated a complete regression of the pathology and symptoms.

## 4. Discussion

The real incidence of OS is still unknown, but according to the most recent publications, it varies from 10% to 40% of chronic bacterial maxillary infections [16]. The most frequent cause of OS is dentoalveolar oral surgery or odontogenic infection with rupture of the Schneiderian membrane and secondary maxillary sinus invasion of the abscess which can invade the other paranasal sinuses. However, OS is often treated by curing the odontogenic pathology and with drainage and surgery of the paranasal sinuses [17].

### 4.1. Correlation Between Quality of Life and OS

According to Gaudin et al., OS was correlated with a statistically significant low-health-related quality of life (QoL), similar to severe chronic pathologies such as diabetes, heart disease, and obstructive pulmonary disease. Furthermore, OS was related to a significantly low-general-health-related QoL compared to non-OS. Early diagnosis and treatment may therefore help to prevent detriment to QoL due to OS [18].

Simuntis et al., who compared SNOT-22 scores between OS and no-OS patients, reported that sleep and functional disorders were more severe in non-odontogenic patients while emotional disturbance was in OS patients, despite several similarities in chronic sinusitis types. Furthermore, malodor is the most significant symptom of odontogenic sinusitis [19].

Cascio et al. described the dislocation of a dental implant in the ethmoidal sinus without causing mucus stagnation and consequent sinusitis. In this case, the presence of the bilateral accessory maxillary orifice provided an additional way for the drainage of mucus in the maxillary sinus, avoiding sinusitis [20].

### 4.2. Correlation Between Anatomical Nose Variations and Maxillary Sinus Fungal Ball

The correlation between anatomical nose variations and OS is more broadly described in the literature; however, Jeon Gang Doo et al. described the predisposing odontogenic and anatomical factors in unilateral maxillary sinus fungal ball (MSFB). They reported that unilateral MSFB was significantly correlated with ipsilateral odontogenic factors and anatomic alteration near the osteomeatal unit, including posterior NSD towards the non-affected side as well as the absence of CB and infraorbital cells [21].

For Sahin et al., the MSFB was more frequent on the concave side of the deviated septum. Furthermore, dental pathologies or the presence of odontogenic treatment history were associated with MSFB [22].

Considering that the fungus ball could be a consequence of a poorly treated OS, the result obtained by Jeon Gang Doo et al. and Sahin et al. can be compared to the present study. For Jeon Gang Doo and Sahin, it remains unclear whether the concave side of the NSD is a predisposing factor for MSFB, and further studies are needed. These results are in contrast with those presented in this manuscript.

In our sample, the combination of significant septal deviations and large chonchae bullosa on the side of sinusitis are the two anomalies that most impact the ventilation of the maxillary sinus, causing stagnation of secretions. Furthermore, these patients had a reduction in their QoL caused by a reduction in the quality of sleep, increased irritability, reduced concentration, and, inevitably, a reduction in productivity.

### 4.3. Correlation Between Anatomical Nose Variations and LMS

Another result obtained in this study is the lack of correlation between the anatomical variables and LMS. The reason is unclear, but it could depend on the fact that the different degrees of opacity of paranasal sinuses are correlated with the time of CT scans and the beginning of the infection, therefore increasing with a late diagnosis. For Lei Liu et al., anatomical variants were common in chronic rino-sinusitis (CRS) and possibly correlated with local CRS but not with diffused CRS [23]; this should be the cause of an uncorrelated LMS. For Niknami et al., the correlation between anatomical variations and the opacification of sinuses is not clear, and only PMT is correlated with a higher LMS in the maxillary sinus [24].

Kaygusuz et al. calculated and compared the LMS of the maxillary, ethmoid, and frontal sinus to the rate of septal deviation, concha bullosa, and agger nasi cells. No significant correlation was found (*p* > 0.05). The results of the study showed no statistically significant correlation between sinonasal anatomical variations and pathologies of the paranasal sinus [25].

Bhagat et al. reported that the incidence of anatomical variations in patients with CRS was similar to that in other studies conducted in asymptomatic populations. Therefore, the sole detection of a solitary anatomical variant itself does not determine predisposition to disease or the pathogenesis of CRS. Regarding surgical management, a critical approach toward anatomical variations should be considered [26].

According to the results of this study and those similarly presented above, a correlation between total LMS and anatomical variation is not detected, while a singular anatomical variation could increase the LMS of a single sinus.

### 4.4. Correlation Between Quality of Life and LMS

Also, there is no correlation between LMS and the severity of symptoms. According to Schalek et al. [27], the CT score has no linear relationship with the QoL preoperatively and cannot serve as a predictor for the outcomes of surgical treatment. For Hopkins et al., there was no correlation between LMS and SNOT-22; the LMS measures a different aspect of the disease according to “subjective” symptom scores. However, it is related to other characteristics of disease severity, the kind of surgery, and its outcome (complication and revision rate) [28,29,30,31].

### 4.5. Limits of This Study and Future Prospective Studies

Our study is characterized by a few limitations. This is a double-center study with a retrospective design and a relatively small number of patients included. In this study, we analyzed only patients who underwent ESS, which could have led to selection bias. An analysis of the difference in the distribution of sinonasal anatomical variants in patients who underwent EES vs. those who underwent conservative treatment could be an interesting topic for future studies (control group). Although numerous anatomical variations exist, only NSD, CB, and PMT on the same side as OS were analyzed. Furthermore, NSD has different characteristics (anterior, posterior, degree of angle, total, and partial) and we did not analyze them in detail. For greater scientific rigor, future studies are needed with analysis of other anatomical variants (Haller Cells, Unicinate Bulla, infraorbital ethmoid cells, maxillary underwood septum, etc.) or with more details on the type of NSD (using the Mladina or Baumann Classification [32]) or the form/shape of the maxillary sinus. Rhinomanometric studies that can accurately reflect the degree of patency of the outflow tract should be performed. Therefore, prospective studies including a sample of adequate size, with anonymous questionnaires, are needed in order to establish a correlation between QoL and degree of CT opacification with anatomical variables in OS; these should also include the possibility of analyzing other anatomical anomalies in greater detail. In the literature, differences in nasal anatomical variants regarding sex are often described, but further studies would be interesting [33].

### 4.6. Author Recommendation Guide in Cases of OS

The authors’ recommended steps in cases of OS are as follows:

- Medical therapy to treat the infection;
- Dental visit with treatment of the dental problem;
- Wait for about 1 month and see if OS regresses or if there are relapses of the pathology;
- In cases of failure of dental and medical therapy or in cases of recurrence of the disease, endoscopic nasal surgery (ESS) is always recommended;
- Multidisciplinary collaboration is mandatory.

Ultimately, this study strengthened evidence on the correlation between a reduction in QoL and the presence of NSD with CB in OS, underlining the importance of a pre-surgery plan to treat not only the sinus problem but also to correct anatomical anomalies.

## 5. Conclusions

This study explored the correlation between certain nasal anatomical variables and SNOT-22, in particular, a combination between NSD and CB or PMT, on the same side as the disease, and it showed a significant correlation with a worse QoL in OS.

Regarding the radiological features of ODS, no significant correlation was found between the LMS and anatomical variations. Other studies or meta-analyses are needed to better analyze these variables and further clarify these results.

## Figures and Tables

**Table 1:** Study population characteristics.

	Population N (%); M ± SD
**Gender**	
**Male**	45/70 (64.3%)
**Female**	25/70 (35.7%)
**Age**	48.15 ± 12.84
**SNOT-22**	48.1 ± 20.1
**SNOT-8**	26.3 ± 8.5
**LMS**	5.1 ± 1.6
**Surgery**	
**Maxillectomy**	70 (100%)
**Ethmoidectomy**	32/70 (45.7%)
**Frontal senectomy**	25/70 (35.7%)
**Septoplasty**	32/70 (45.7%)
**Plastic of Middle Turbinate**	53/70 (75.7%)
**Tooth extraxtion**	42/70 (60%)

*N* Number; *M* Media; *SD* Standard Deviation; *LMS* Lund-Mackay Score.

**Table 2:** Logistical regression between SNOT-22 and anatomical nasal variations.

	N (%)	M (±SD) SNOT 22	Odd Ratio	95% Confidence Interval	*p* Value
**NSD isolated**	16 (22.8%)	53.25 (±6.9)	1.01	0.97–1.06	0.42
**CB or PMT isolated**	24 (34.2%)	45.33 (±16.3)	0.98	0.95–1.02	0.98
**NSD with CB or PMT**	16 (22.8%)	71.12 (±10.1)	1.34	1.03–1.73	0.02

*N*—number; *M*—media; *SD*—standard deviation; *NSD*—nasal septum deviation; *CB*—concha bullosa; *PMT*—paradoxical middle turbinate.

**Table 3:** Logistical Regression between SNOT-8 and anatomical nasal variations.

	N (%)	M (±SD) SNOT-8	Odd Ratio	95% Confidence Interval	*p* Value
**NSD isolated**	16 (22.8%)	28.62 (±4.4)	1.04	0.94–1.15	0.39
**CB or PMT isolated**	24 (34.2%)	27.33 (±7.1)	1.02	0.93–1.11	0.61
**NSD and CB or PMT**	16 (22.8%)	32.25 (±7.5)	1.17	1.00–1.37	0.04

*N*—number; *M*—media; *SD*—standard deviation; *NSD*—nasal septum deviation; *CB*—concha bullosa; *PMT*—paradoxical middle turbinate.

**Table 4:** Logistical regression between Lund–Mackay Score and anatomical nasal variations.

	N (%)	M (±SD) LMS	Odd Ratio	95% Confidence Interval	*p* Value
**NSD isolated**	16 (22.8%)	5.05 (±1.6)	1.41	0.86–2.32	0.16
**CB or PMT isolated**	24 (34.2%)	5.75 (±1.1)	1.03	1.06–1.56	0.88
**NSD and CB or PMT**	16 (22.8%)	6 (±2.1)	1.15	0.86–2.61	0.12

*N*—number; *M*—media; *SD*—standard deviation; *NSD*—nasal septum deviation; *CB*—concha bullosa; *PMT*—paradoxical middle turbinate.

## Data Availability

No new data were created or analyzed in this study.
